# Poorly differentiated mucinous carcinoma of the ascending colon complicated by bilateral ovarian mature cystic teratomas in a 17-year-old female patient: a case report

**DOI:** 10.1186/s40792-024-01892-z

**Published:** 2024-04-23

**Authors:** Takuya Shimogawa, Yukiharu Hiyoshi, Mayuko Ouchi, Keisuke Kosumi, Kojiro Eto, Satoshi Ida, Masaaki Iwatsuki, Yoshifumi Baba, Yuji Miyamoto, Nasa Okazaki, Yuko Miyasato, Hideo Baba

**Affiliations:** 1 Department of Gastroenterological Surgery, Graduate School of Medical Sciences, Kumamoto University, 1-1-1 Honjo, Kumamoto, 860-8556 Japan; 2 Department of Diagnostic Pathology, Kumamoto University Hospital, 1-1-1 Honjo, Kumamoto, 860-8556 Japan

**Keywords:** Colorectal cancer, Adolescents and young adults, Ovarian tumor, Mature cystic teratoma, Fertility, Adjuvant therapy

## Abstract

**Background:**

Colorectal cancer (CRC) is one of the most common cancers worldwide, and screening colonoscopy has led to a decreasing incidence rate. However, the incidence of CRC is increasing among young people, especially adolescents and young adults (AYAs) who are not routinely screened. Although CRC is the fourth most common cancer among AYAs, it is extremely rare. In younger patients, CRC is often diagnosed later, and the proportion of patients with advanced CRC is higher than that in older patients. We herein present a case of poorly differentiated mucinous carcinoma of the ascending colon complicated by bilateral ovarian mature cystic teratomas (MCTs) in an AYA.

**Case presentation:**

A 17-year-old female patient presented with a chief complaint of abdominal pain and diarrhea that had persisted for more than 3 years. Colonoscopy revealed circumferential wall thickening of the ascending colon, and colonic biopsy revealed a mucous mass and findings of adenocarcinoma, predominantly signet ring cell carcinoma. Abdominal computed tomography (CT) and pelvic magnetic resonance imaging (MRI) showed bilateral ovarian tumors. Laparoscopic right hemicolectomy and enucleation of bilateral ovarian tumors were performed. Although the ascending colon cancer formed a large mass, there were no signs of peritoneal dissemination or direct invasion to the surrounding organs. Microscopically, the ascending colon was a poorly differentiated mucinous carcinoma with signet ring cell carcinoma and lymph node metastasis (9/42). The ovarian tumors were diagnosed as MCTs without any malignant components. The pathological diagnosis was ascending colon cancer (pT4aN2bM0, pStage IIIC) and bilateral ovarian MCTs. Microsatellite instability (MSI) testing was negative, and there were no gene mutations in either RAS or BRAF. Postoperative adjuvant chemotherapy with oxaliplatin and 5-FU was started.

**Conclusions:**

We presented a case of locally advanced ascending colon cancer in a 17-year-old female patient. CRC rarely occurs in AYAs. However, the incidence has gradually increased in recent years. It should be considered as a differential diagnosis for young patients with long-term abdominal symptoms of unknown cause.

## Introduction

Colorectal cancer (CRC) affects more than 1.9 million people worldwide each year and causes approximately 935,000 deaths. Among all malignant tumors, it is the third most common cause of morbidity and the second most common cause of death [1]. The incidence of CRC has declined over the past few years, possibly due to colonoscopy screening in patients aged over 50 years of age [2]. In contrast, the incidence of CRC among patients younger than 50 years of age has increased by 1.5% and 1.6% in males and females, respectively. In Japan, approximately one million people are diagnosed with cancer annually, of whom 2% are adolescents and young adults (AYAs: age 15–39 years) [3]. Among the Japanese AYA population, CRC is the fourth most common cancer after breast, uterine, and thyroid cancers [4]. As a result, CRC screening is not recommended for healthy AYAs. In this background it is difficult to diagnose CRC at an asymptomatic stage in this age group. Additionally, failure to recognize or deny the importance of symptoms may lead to a delayed diagnosis [5].

We herein report the case of a 17-year-old female patient with poorly differentiated mucinous carcinoma of the ascending colon which was complicated by bilateral ovarian mature cystic teratomas (MCTs). To the best of our knowledge, this is the first report of colon cancer complicated by bilateral MCTs.

## Case presentation

**Fig. 1:**
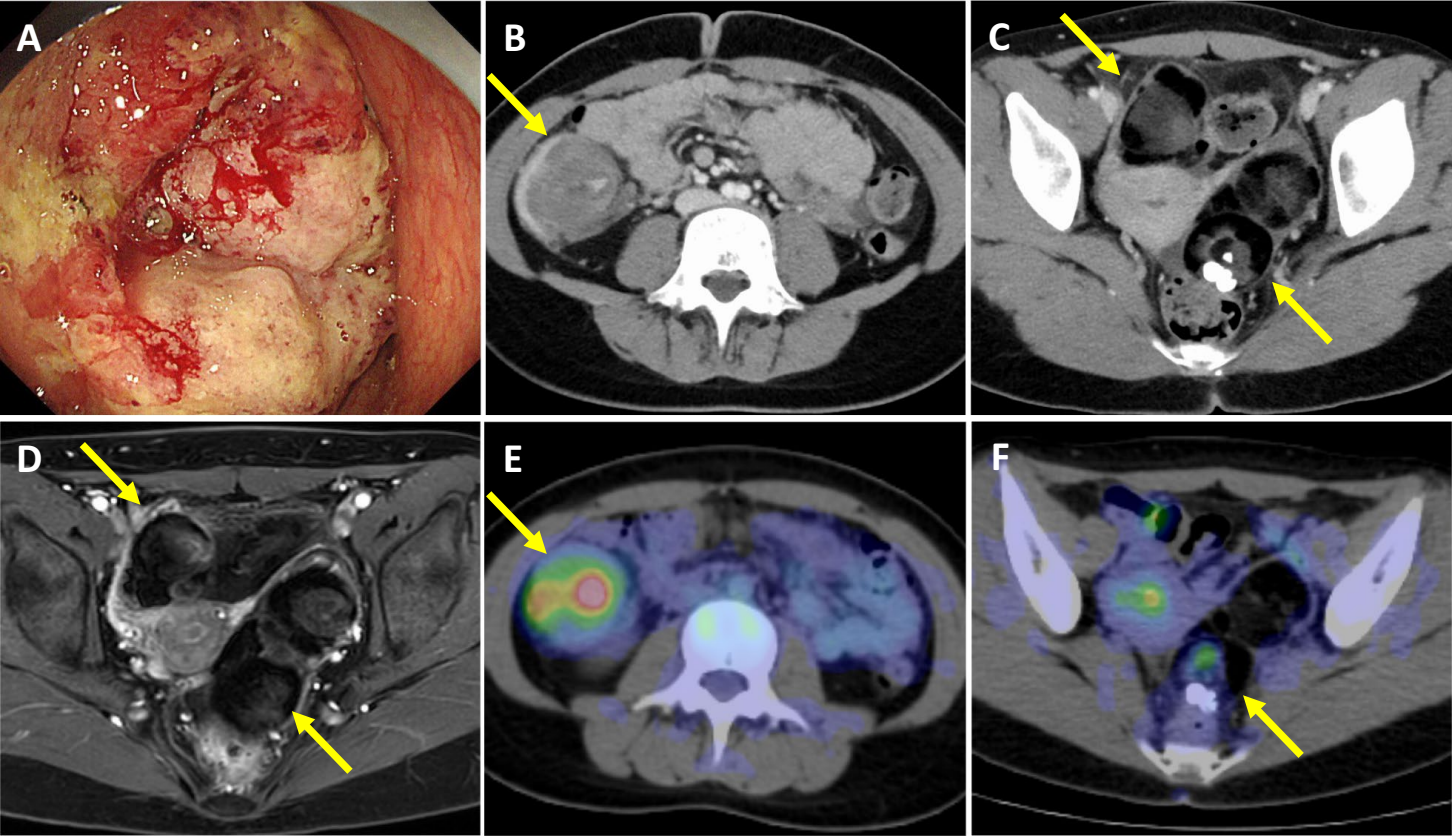
Preoperative images of ascending colon cancer and bilateral ovarian tumors. **A** Colonoscopy revealed circumferential wall thickening of the ascending colon, which was difficult to pass with the scope. **B**, **C** Contrast-enhanced CT showed wall thickening of the ascending colon (**B**; arrow) and bilateral ovarian tumors (C; arrows). **D** Pelvic MRI showed bilateral ovarian tumors (arrows). **E**, **F** FDG–PET showed the increased uptake of FDG at the ascending colon (E; arrow) and bilateral ovarian tumors (**F**; arrows)

A 17-year-old female patient presented with a chief complaint of abdominal pain and diarrhea. These symptoms had continued intermittently for about 3 years, since she was 14 years of age, and she had seen a local doctor, but she had never had an imaging study. The patient was healthy, had no history of either hereditary or inflammatory diseases, and no family history of cancer. Screening abdominal ultrasonography revealed thickening of the wall of the ascending colon and dilatation of the small intestine. Colonoscopy revealed circumferential wall thickening of the ascending colon; however, the scope could not be inserted into the ileum due to obstruction of the lumen (Fig. 1A). A colonic biopsy revealed a mucous mass and adenocarcinoma, predominantly signet ring cell carcinoma. Abdominal computed tomography (CT) showed wall thickening and fluid collection in the ascending colon, with swollen lymph nodes (LNs), raising the suspicion of LN metastasis (Fig. 1B). In addition, a soft tissue shadow with calcification and fat was found on the left dorsal side and right ventral side of the uterus (Fig. 1C). Pelvic magnetic resonance imaging (MRI) showed masses of approximately 50 mm on the right side and 80 mm on the left side of the pelvis, with multinodular fatty components and some substantial components, and a contrast effect, suggesting bilateral ovarian teratomas (Fig. 1D). Fluorodeoxyglucose–positron emission tomography (FDG–PET) showed the increased uptake of FDG in the ascending colon [standardized uptake value (SUV) max: 7.8], a soft tissue shadow on the left dorsal uterus [SUVmax: 3.1], and a soft tissue shadow on the right ventral side of the uterus [SUVmax: 2.6] (Fig. 1E and F). Blood tests showed that the patient’s tumor marker levels were high (CEA, 3.7 ng/ml; CA19-9, 37.0 U/ml). A preoperative diagnosis of advanced ascending colon cancer with LN metastasis and bilateral ovarian teratomas was made, and laparoscopic right hemicolectomy and enucleation of bilateral ovarian tumors were performed.

**Fig. 2:**
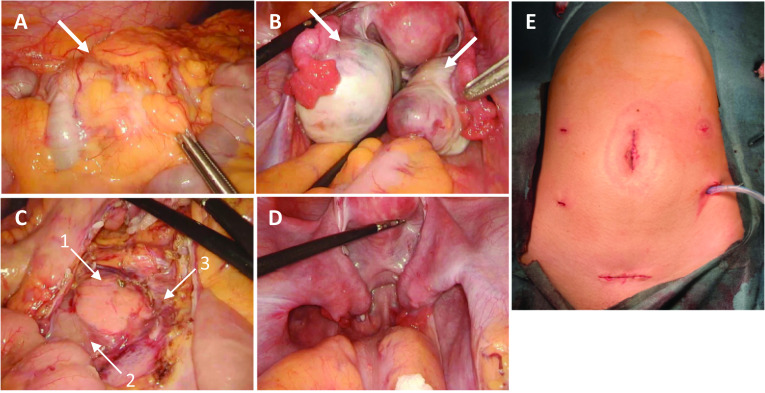
Intraoperative images. Intraoperative images of ascending colon cancer (**A**; arrow) and bilateral ovarian tumors (**B**; arrows). Images obtained after right hemicolectomy (**C**) [pancreas (arrow 1), duodenum (arrow 2), superior mesenteric vein (arrow 3)]. Images obtained after enucleation of bilateral ovarian tumors (**D**). **E** Postoperative wounds and drain

A port was placed in the umbilical incision and insufflation was performed to observe the intra-abdominal cavity. An enlarged tumor in the ascending colon and bilateral ovarian tumors were identified (Fig. 2A, B). The ascites cytology of the Douglas fossa was negative. A right hemicolectomy and D3 lymph node dissection were performed (Fig. 2C). Bowel reconstruction was performed extracorporeally using functional end-to-end anastomosis (FEEA). Bilateral ovarian tumor enucleation was then performed (Fig. 2D), and the ascending colon and ovarian tumors were removed through a suprapubic transverse incision (Fig. 2E).

**Fig. 3:**
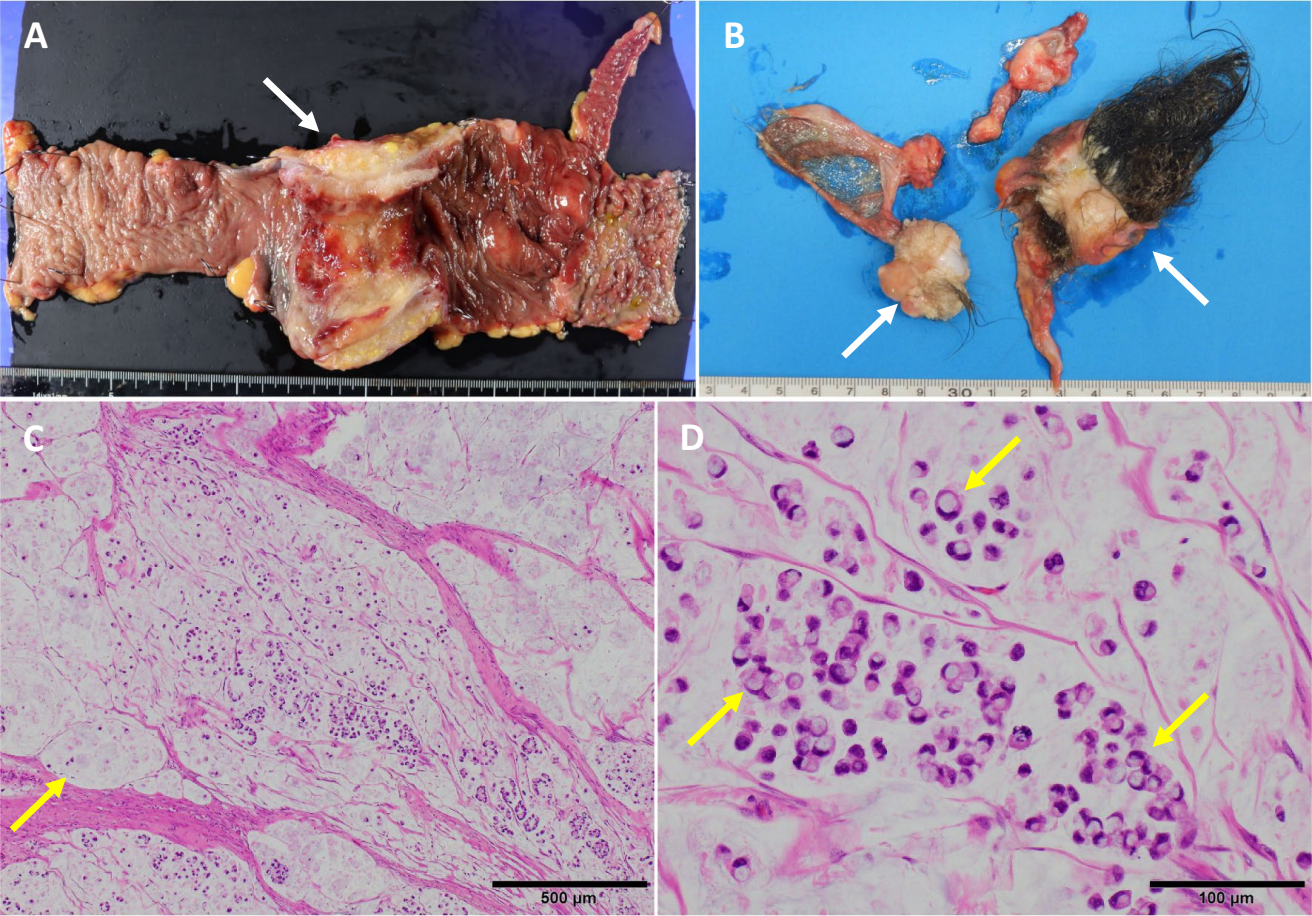
Image of resected specimen. **A** 5-cm-long region of circumferential wall thickening is observed in the ascending colon (arrow). **B** Bilateral ovarian tumors had lumens filled with hair and fat (arrows). **C** Microscopically, the tumor forms a mucous lake (arrow). **D** Pathological examination revealed poorly differentiated mucinous carcinoma formed by the extracellular secretion of large amounts of mucus from signet ring cell carcinoma (arrows)

Grossly, 25 cm of the ascending colon and 5 cm of the ileum (Fig. 3A) and bilateral ovarian tumors were removed (Fig. 3B). A 5-cm-long region of circumferential wall thickening was observed in the ascending colon. The bilateral ovarian tumors had lumens filled with hair and fat. Microscopically, the tumor formed a mucous lake (Fig. 3C), and a pathological examination revealed poorly differentiated mucinous carcinoma with signet ring cells (Fig. 3D) and a small coexisting component of highly differentiated mucinous carcinoma, including tubular adenocarcinoma. The carcinoma was exposed on the serosal surface. Forty-two lymph nodes were dissected, and nine metastatic lymph nodes, including five paraintestinal lymph nodes and four intermediate lymph nodes. The ovarian tumors were diagnosed as MCTs with no coexisting malignant component, as both sides showed skin-like nodules, hair follicles, and sweat glands.

The patient’s postoperative course was uneventful. Oral intake was initiated on the third postoperative day and the patient was discharged on the seventh postoperative day. The pathological diagnosis was pT4aN2bM0, stage IIIC. Microsatellite instability (MSI) testing was negative, and both RAS and BRAF were wild type. Postoperative adjuvant chemotherapy with oxaliplatin and 5-FU was initiated with a planned duration of 6 months.

## Discussion

The present report describes a case of poorly differentiated mucinous carcinoma of the ascending colon complicated by bilateral ovarian MCTs in an AYA. The term AYA refers to the generation between childhood and middle age. The age and definition of are not consistent among countries and academic societies [6]. Epidemiological data on cancer in AYAs are more limited in comparison to data on cancer in children and adults. Although CRC is the fourth most common cancer among AYAs, it is a sporadic disease among Japanese individuals of 15–19 year age with an estimated incidence rate of 1.2 per million population [4].

Hereditary factors have been reported to primarily influence CRC in young patients [7]. However, most CRCs among AYAs, which have been on the rise in recent years, are sporadic rather than hereditary [8]. Several social and behavioral risk factors, such as alcohol consumption, tobacco smoking, red meat intake, and obesity are associated with CRC [9]. The present case had no family history of CRC, and the MSI test results were negative. The patient also had no history of alcohol consumption or smoking and was not obese, so the above risk factors were not considered to have affected carcinogenesis in this case. Therefore, this case may be a sporadic carcinoma from other causes or carcinogenesis due to an unknown genetic abnormality associated with CRC.

The symptoms of CRC in AYAs are often nonspecific and include vague abdominal pain and weight loss. AYAs tend to wait longer to be diagnosed due to the low suspicion of malignancy at this age. Advanced stage at presentation is more likely in AYAs than in older adults with CRC, perhaps due to diagnostic delay and lack of screening [10]. This patient had been visiting a local doctor for 3 years with complaints of abdominal pain and diarrhea, but the cause remained unknown. This case was referred to our hospital by a local doctor because Crohn's disease was suspected due to the persistence of these symptoms. If CRC had been considered as a differential diagnosis and CT or colonoscopy had been performed to investigate her symptoms, this case might have been diagnosed at an earlier stage.

Among AYAs, CRC of the rectum and left-sided colon is more common than CRC of the right-sided colon [9]. In contrast, hereditary CRC, such as Lynch syndrome, is characterized by its tendency to be located in the right colon [8]. Although this case involved right-sided colon cancer, hereditary CRC such as Lynch syndrome was not suspected because she had no family history of cancer and was microsatellite stable. CRC among AYAs is also pathologically characterized by a high percentage of mucinous and signet-ring cell carcinomas [7]. A pathological examination revealed poorly differentiated mucinous carcinoma with signet ring cells, which is consistent with the features of CRC among the AYAs. Long-term survival rates for CRC are improving in both AYAs and older adults. Whether the prognosis is different for young patients is controversial, but younger patients are more likely to receive systemic therapy at any stage of the disease, suggesting similar or better outcomes than older patients [10]. Signet ring histology was associated with lower relative survival, stage-for-stage, in comparison to mucinous or non-mucinous non-signet ring adenocarcinomas of the colon. Among patients with tumors in the colon, the relative survival for those with mucinous cancers was not different from that of patients with non-mucinous, non-signet adenocarcinomas [11]. The pathology of this case was mucinous carcinoma, but it was a poorly differentiated type of mucinous carcinoma, including signet-ring cell carcinoma. Therefore, we initiated adjuvant therapy considering that the grade of the disease was the same as that of signet ring cell carcinoma.

Fertility is also a significant issue for AYAs. Our patient had fertility issues during adjuvant therapy for colon cancer. The total fertility rate has been reported to be lower in female CRC survivors than in the general population [12]. She was a young woman, and her family wished to preserve her fertility. The risk of chemotherapy-induced infertility depends on the chemotherapy regimen [13, 14]. The clinical guidelines do not consider CRC among the AYAs as a criterion to drive treatment, either in an adjuvant or metastatic setting [15]. The Japanese CRC guidelines recommend adjuvant therapy for Stage III CRC, and the regimen is 5-fluorouracil (5-FU) alone or in combination with oxaliplatin. 5-FU carries a low risk of amenorrhea in female patients, whereas oxaliplatin has been suggested to have moderate gonadal toxicity [13, 14]. Before starting adjuvant therapy, we referred the patient to a fertility preservation specialist. We used 5-FU combined with oxaliplatin for adjuvant therapy because the ovarian dysfunction caused by these adjuvant therapies is considered to be temporary.

In this case, CT incidentally revealed bilateral ovarian tumors. Since the patient had colon cancer, we first considered ovarian metastasis. However, the preoperative diagnosis was MCT because the CT and MRI results showed a multifocal tumor with fatty components and calcification. MCT is the most frequent ovarian germ cell tumor, accounting for 20% of all ovarian tumors. Only 10% of MCTs are bilateral. Malignant transformation of MCT can occur in 1–3% of cases. The most common malignancy arising from teratomas is squamous cell carcinoma (SCC) of ectodermal origin, representing up to 85% of all cases [16]. However, to our knowledge, this is the first case report of simultaneous MCT and malignant tumors. This case of complicated CRC due to MCT was considered incidental at this time.

## Conclusions

CRC in AYAs is rare, but the incidence has gradually increased in recent years. Since AYAs are more likely to be diagnosed with advanced cancer than the elderly, it is essential to detect the disease as early as possible and to provide treatment. For young patients with symptoms such as prolonged abdominal pain of unknown cause, CRC may be considered as a differential diagnosis, and screening tests such as colonoscopy should be considered.

## Data Availability

All data generated or analyzed during this study are included in this published article.

## Abbreviations

CRC: Colorectal cancer
AYA: Adolescents and young adults
MCT: Mature cystic teratoma
CT: Computed tomography
MRI: Magnetic resonance imaging
MSI: Microsatellite instability
LN: Lymph node
FDG–PET: Fluorodeoxyglucose–positron emission tomography
SUV: Standardized uptake value
FEEA: Functional end-to-end anastomosis
5-FU: 5-Fluorouracil
SCC: Squamous cell carcinoma
